# Supplementary material for: Preparation of Porous Liquid Based on Silicalite-1
Source: Materials (Basel). 2019 Dec 1;12(23):3984. doi: 10.3390/ma12233984 (PMC6926578; doi:10.3390/ma12233984)
Supplement: Supplementary file 1 [file materials-12-03984-s001.pdf]

Supporting Information

Preparation of Porous Liquid based on Silicalite-1

Yutong Liu [1,2], Yang Bai [1] and Tao Tian [1,*]

[1] Key Laboratory of Groundwater Resources and Environment, Ministry of Education, College of Environment and Resource, Jilin University, Changchun 130012, China; tiantao@jlu.edu.cn
[2] Jilin Engineering Normal University; liuyt841011@163.com
* Correspondence: tiantao@jlu.edu.cn

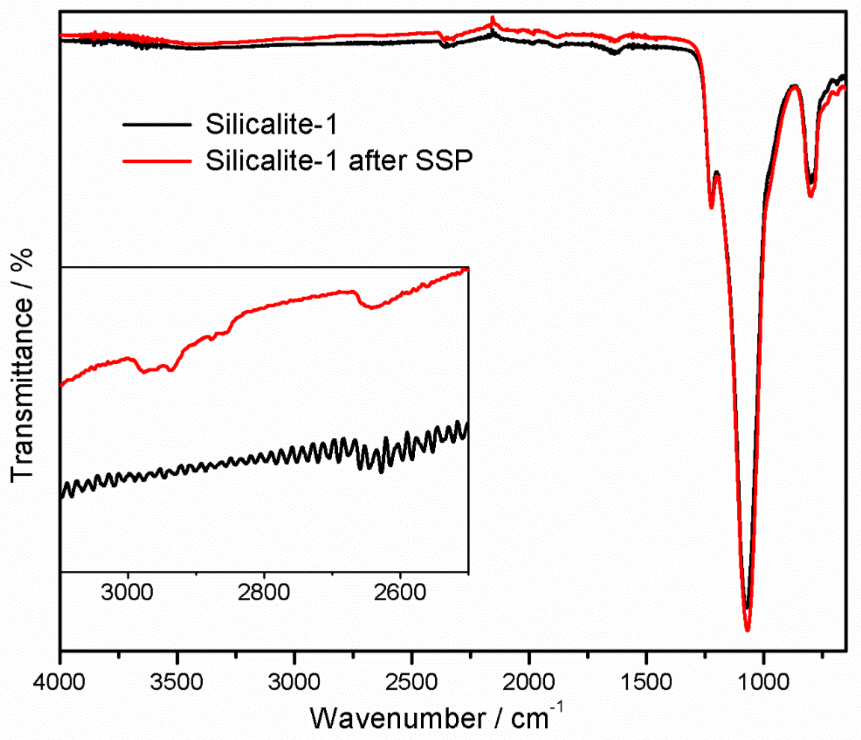

Figure S1. FT-IR spectra of S-1 and S-1 treated by SSP.

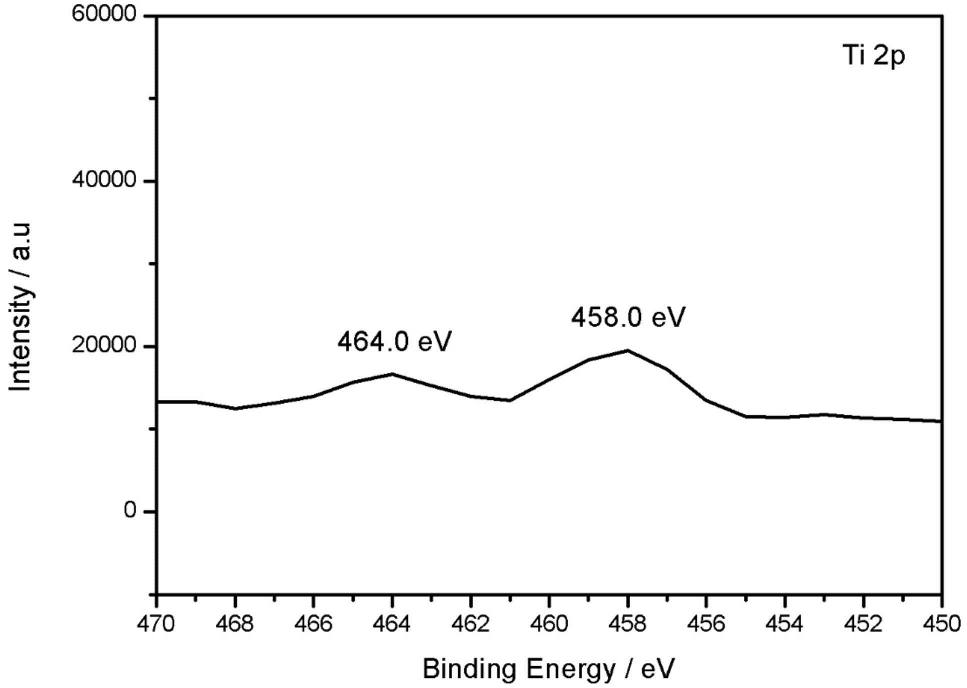

Figure S2. XPS spectra of the P_2p region of S-1 treated by SSP.

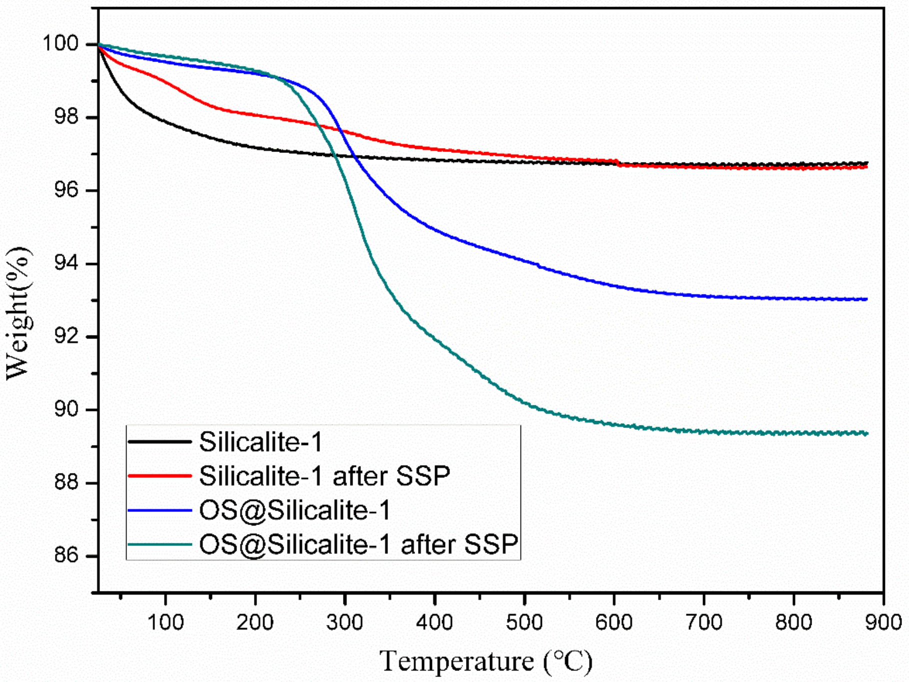

Figure S3. TGA trace of S-1, S-1 after SSP, OS@S-1 and OS@S-1 after SSP.

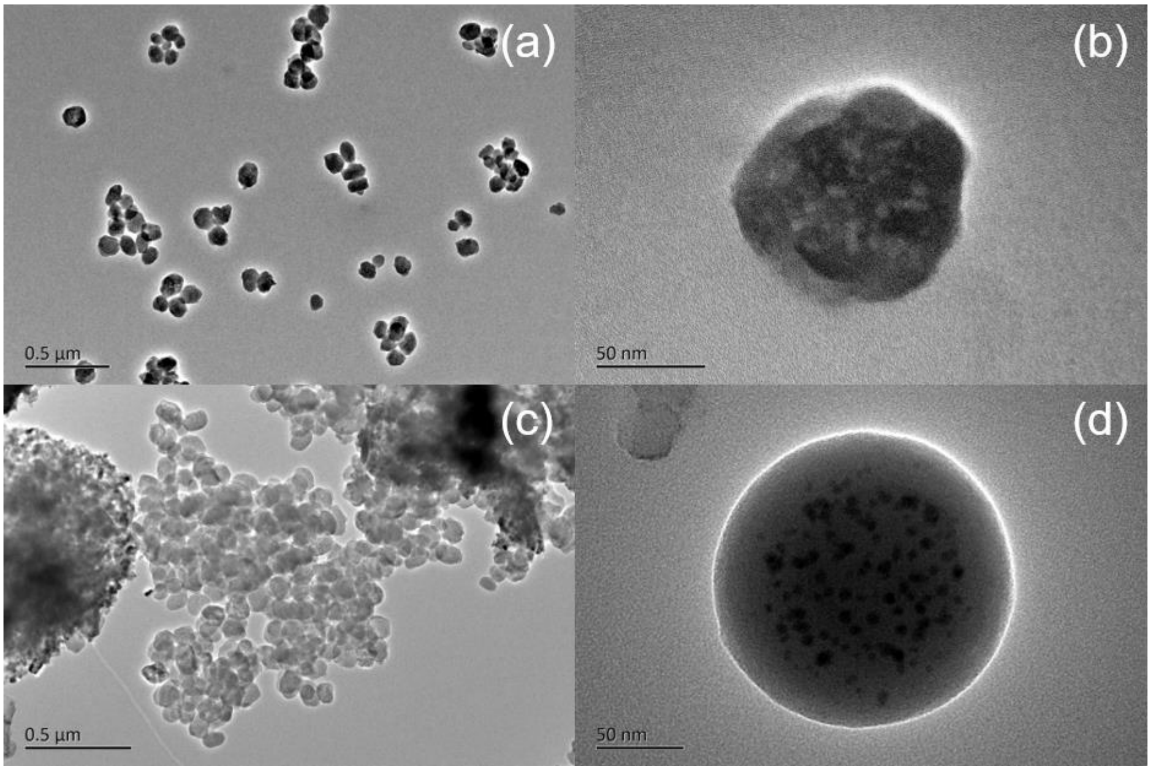

Figure S4. TEM images of S-1 (a, b) and OS@S-1 (c, d).
